# Pharmacokinetic changes for newer antiepileptic drugs and seizure control during pregnancy

**DOI:** 10.1111/cns.13796

**Published:** 2022-01-17

**Authors:** Xiaotong Yin, Yan Liu, Yang Guo, Limei Zhao, Guofei Li, Xiaoping Tan

**Affiliations:** ^1^ Department of Neurology Shengjing Hospital of China Medical University Shenyang Liaoning PR China; ^2^ Department of Pharmacy Shengjing Hospital of China Medical University Shenyang Liaoning PR China

**Keywords:** antiepileptic drugs, pharmacokinetics, seizure frequency, women with epilepsy

## Abstract

**Objective:**

To investigate pharmacokinetic changes in newer antiepileptic drugs (AEDs) and assess seizure frequencies and risk factors of increased seizures during pregnancy in women with epilepsy (WWE).

**Methods:**

A total of 56 pregnancies in 53 WWE who received newer antiepileptic drugs (AEDs) were enrolled. Data on seizure activity and types, daily dose, and AEDs blood levels were derived from routine clinical follow‐up. Changes in AEDs clearance were compared between each trimester and nonpregnant baseline. The ratio of AED levels of each trimester to their targets (nonpregnant baseline) concentrations (RTC) was compared between patients with and without an increased seizure. A binary logistic regression was used to investigate the risk factors contributing to seizure worsening during pregnancy.

**Results:**

Increased clearances of LTG, LEV, and OXC were observed in all trimesters versus nonpregnant baseline. The peak changes in the clearance of LTG (3.42‐fold baseline clearance) (*p *< 0.001) and LEV (2.78‐fold) (*p *< 0.001) occurred in the second trimester during pregnancy, followed by oxcarbazepine (2.11‐fold) in the third trimester (*p *< 0.03). Plasma concentrations of LTG and LEV during pregnancy were significantly decreased compared to baseline levels, except for OXC. However, no significant differences in RTC values were observed between patients with and without seizure worsening. Some risk factors as seizures for the prior nine months could significantly affect seizure frequency during pregnancy.

**Conclusion:**

We found substantial changes in the pharmacokinetics of multiple newer AEDs in WWE, reinforcing the need for therapeutic drug monitoring (TDM) during pregnancy. We would encourage at least one monitoring every trimester and probably more frequently for women with poorly seizure control before pregnancy, and AEDs dose adjustment should keep up with clearance changes. In addition, a well‐controlled seizure nine months before pregnancy could lower the risks of seizure during pregnancy, highlighting the importance of pre‐pregnancy counseling and seizure management before pregnancy.

## Graphical abstract

Substantial changes in the pharmacokinetics of multiple newer AEDs during pregnancy reinforce the need for therapeutic drug monitoring (TDM). We would encourage at least one monitoring every trimester and probably more frequently for women with poorly seizure control before pregnancy, and AEDs dose adjustment should keep up with clearance changes to lower the risks of seizure during pregnancy.

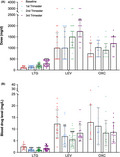

## 1 INTRODUCTION

Epilepsy is a common neurological problem in the patient population, which presents a unique management challenge for women with epilepsy (WWE) during pregnancy, controlling the risks of increased seizure frequency to maternal and fetal during pregnancy and lowering fetal exposure to antiepileptic drugs (AEDs). Given that the first generation of AEDs may produce adverse events such as congenital malformations and severe cognitive dysfunction for offspring,^1^ the newer AEDs, characterized by low teratogenic rate and well seizure control, have been widely used in women of childbearing age and WWE during pregnancy in recent years.^2^ However, during pregnancy and postpartum, increased plasma volume of distribution, elevated renal clearance, and the induction of liver metabolism contribute to marked pharmacokinetic changes in many of AEDs, particularly in newer AEDs as lamotrigine (LTG), the magnitude of clearance changes of which exceeds many of the older AEDs,^3^, ^4^ making it challenging to maintain a stable blood level during pregnancy.^4^, ^5^ These changes may affect the seizure frequency during pregnancy and the fetal exposure to AEDs. Therefore, understanding the clearance changes in newer AEDs and the extent of fluctuations in blood concentration may help drug dosage adjustments to maintain seizure‐free or keep seizure stability during pregnancy.

Previous studies have shown that pregnancy significantly affects the circulating blood concentration of LTG,^6^ followed by levetiracetam (LEV) and oxcarbazepine (OXC).^5^ The levels of these drugs decrease as pregnancy progresses and probably have a significant inter‐individual variability.^7^ Therapeutic drug monitoring (TDM) may be assisted in adjusting the AEDs dose during pregnancy, although the lack of robust evidence.^8^ Pennell et al. recommended monthly monitoring of AED levels during pregnancy, with the possible exception of carbamazepine (CBZ) due to the clearance and dose being virtually unchanged.^3^ Some studies suggested a dosage adjustment if an AED level decreased by 15%–25% that of the nonpregnant target concentration, especially for pregnancies with risk factors of convulsions.^9^, ^10^ Currently, available data on pharmacokinetics changes correlated with pregnancy and dosage adjustments guided by TDM for newer AEDs are insufficient, especially for LEV and OXC. In this study, we investigated further the changes in both clearance and concentration of these three newer AEDs during pregnancy and whether these alterations were related to the deterioration of seizure control.

## 2 METHODS

### 2.1 Study population

The detailed clinical data of WWE taking newer AEDs during pregnancy admitted to Epilepsy Center of Shengjing Hospital were collected from January 2014 to December 2020. Inclusion criteria were as follows: (1) age≥18; (2) epilepsy diagnosis was under criteria issued by the International League Against Epilepsy—ILAE in 2014; (3) gestational age (GA) <16 weeks; (4) at least one blood AED concentration value and corresponding oral daily dose were available during any pregnant trimesters—nonpregnant baseline, first trimester (<14 weeks), second trimester (14 to 28 weeks), and third trimester (29 to 42 weeks); and (5) follow‐up every 1–3 months. Exclusion criteria were as follows: patients (1) accompanied by cardiopulmonary dysfunction and renal or hepatic dysfunction; (2) with a progressive cerebral disease, significant mental retardation (IQ<70), depression, schizophrenia, or other severe mental disorders; (3) coadministration of medicines known to affect the pregnancy outcome; (4) noncompliance, or inability to keep daily calendars for AED doses, and the types and number of seizures; and (5) who did not allow to clinic data collection or follow‐up. The Ethics Committee for Clinical Trials of Shengjing Hospital approved this study (No. 2021PS106K).

### 2.2 Study design

This is a retrospective study by collecting clinical data of WWE taking monotherapy or non‐interaction polytherapy of LTG, LEV, and OXC, including age at conception, history of pregnancy, seizure type, and seizure frequency for prior nine months and during pregnancy, AEDs use and blood drug levels obtained through routine clinical practice, the time between the last medication and blood draws. In our epilepsy center, values of the AED serum concentrations were actively used for TDM, and AED dose adjustments were based on individual's target concentration in the nonpregnant period and concentration at follow‐up visits, seizure types, seizure frequency, AEDs‐related side effects, and the characteristic of AED clearances during diverse stages of pregnancy. Trough concentration of LTG, LEV, and active metabolite 10‐monohydroxy derivate (MHD) of oxcarbazepine were measured using a high‐performance liquid chromatography method in clinical laboratories. The total concentrations were analyzed because free concentrations were not always available. The blood samples were taken at a steady state of >5 days after the last dose adjustment and a fasting state before the morning dose.

### 2.3 Data analysis

#### 2.3.1 AEDs clearance

Plasma levels of AEDs were used to calculate the relative clearance of each AED at multiple time points during pregnancy. The relative AED clearance (L/24 h) was calculated as the ratio of the daily dose (mg/day) to total plasma drug concentration (mg/L).^11^ Nonpregnant baseline clearance was defined as obtained before pregnancy and, if unavailable, as postpartum values after six weeks of delivery.^12^ Changes in the relative clearance of different AEDs in each trimester versus the nonpregnant baseline levels were compared using single‐factor analysis of variance (ANOVA). All clearance values were logarithm‐transformed (log10) to the assumption of Gaussian distribution. The mean value was calculated when more than one plasma concentration was available within 24 h for a patient.

#### 2.3.2 Therapeutic drug monitoring and seizure frequency

Analysis of TDM or seizure frequency enrolled all monotherapy cases and LEV polytherapy cases combined with LTG or OXC. Furthermore, from the first nine months of pre‐pregnancy to the end of pregnancy, only patients with a complete history of seizures, a baseline blood concentration and at least one concentration for each trimester of pregnancy were included in the analysis. A detailed history of epilepsy and seizures was obtained from the patient's medical record. To determine whether the seizure was worsening or not, we calculated the relative seizure frequency of all types for each trimester, as previously described by Pennell et al.^6^ The relative frequency in each trimester greater than the nonpregnant baseline frequency (nine months before pregnancy) was coded as 1, representing a worsening in seizures; otherwise, the frequency was 0. We also calculated the ratio of AED levels of each trimester to individual target (nonpregnant baseline) concentration (RTC) for each case; for detail, the RTC denotes the mean plasma AED concentration of each trimester divided by the nonpregnant baseline concentration. For each trimester and overall pregnancy, the mean RTC value was compared between pregnancies experiencing worsening seizures versus those with stable or improved seizure frequency using a two‐sample *t* test. We also used a >35% drop in RTC values from baseline levels as a threshold, since its potential association with increased seizures during pregnancy,^5^, ^6^ to compare the incidence of seizure worsening between pregnancies with a >35% decrease in RTC values and those with fewer RTC changes during each trimester via a chi‐square test.

Besides, a binary logistic regression analysis was performed to screen for risk factors affecting epileptic seizures during pregnancy, including the RTC values, maternal age, seizure during 9 months before pregnancy, polytherapy, and the ratio to a baseline (preconception) relative clearance.

#### 2.3.3 Statistical analyses

IBM SPSS Statistics Version 20.0 (IBM Corp., Armonk, NY) was used for data analyses. For continuous variables, normal distribution was assessed using the Kolmogorov‐Smirnov test. A student's t test or ANOVA test was conducted for normal/Gaussian distribution data, otherwise using a Mann‐Whitney test. For discrete variables, statistical analyses were performed using a chi‐square test. A *p* < 0.05 was considered significant.

## 3 RESULTS

**FIGURE 1:**
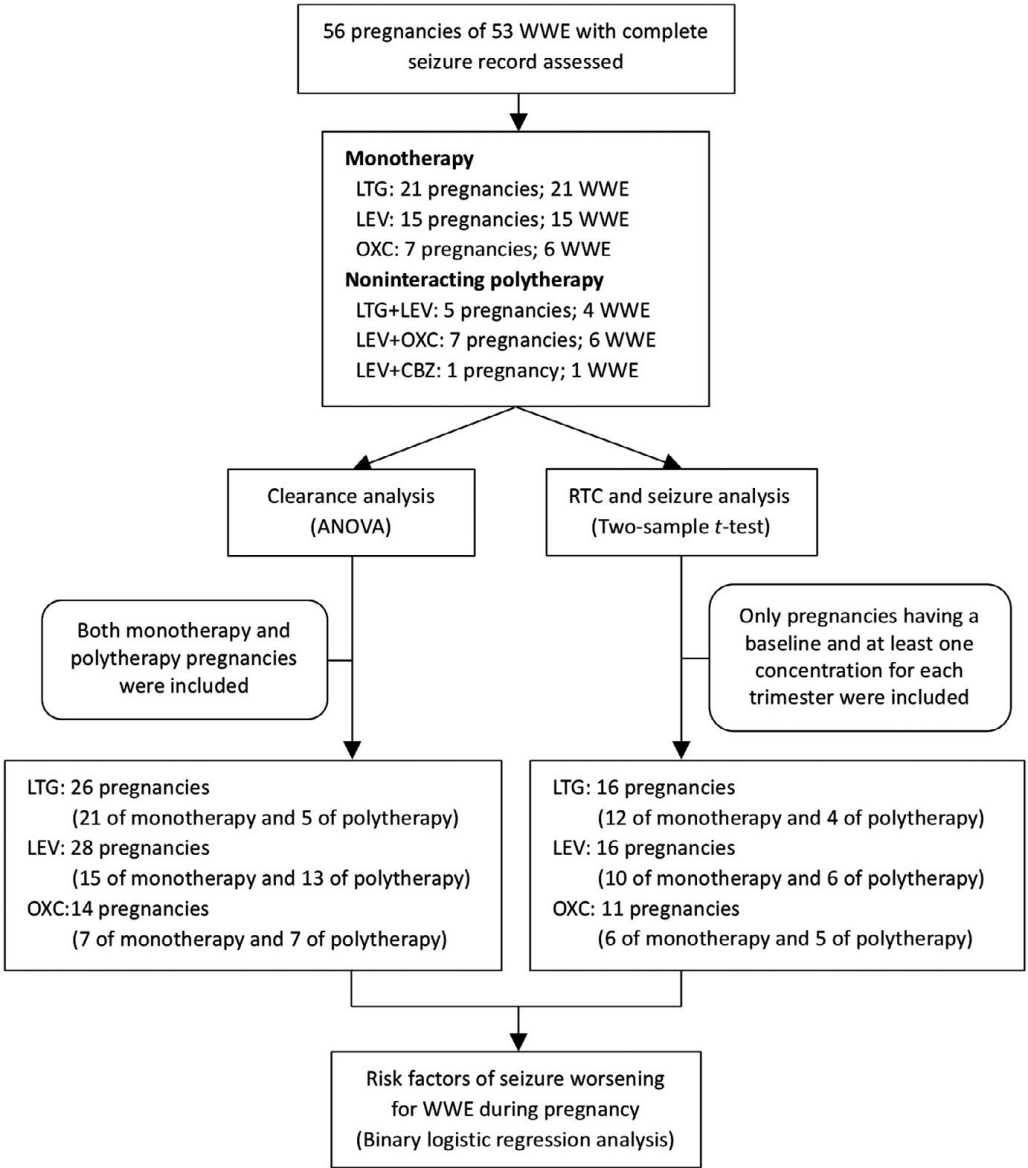
Flow diagram of data analysis and WWE/pregnancies enrolled in the study. ANOVA, single‐factor analysis of variance; CBZ, carbamazepine; LEV, levetiracetam; LTG, lamotrigine; OXC, oxcarbazepine; WWE, women with epilepsy

A flow chart was depicted to show the framework of this work (see Figure 1). A total of 56 pregnancies in 53 WWE and their 387 samples were included in this study (see Figure 1), and 43 pregnancies received monotherapy on LTG, LEV, and OXC, respectively, and 13 pregnancies received non‐interacting AED polytherapy (LEV+LTG, LEV+OXC, LEV+CBZ). All pregnancies with monotherapy and non‐interacting polytherapy were included in the clearance analysis, including 178 samples for LTG from 26 pregnancies, 136 samples for LEV from 28 pregnancies, and 73 samples for OXC from 14 pregnancies. Nevertheless, only those with a baseline drug level and at least one concentration value for each trimester of pregnancy were involved in seizure frequency analysis, including 135 samples for LTG from 16 pregnancies, 92 samples for LEV from16 pregnancies, and 60 samples for OXC from 11 pregnancies. Clinical characteristics of the study population were shown in Table 1.

**TABLE 1:** Clinical characteristics of women with epilepsy (WWE) included in the study

Patients’ characteristics	LTG	LEV	OXC	LTG+LEV	LEV+OXC	LEV+CBZ
Total no. of WWE	21	15	6	4	6	1
Total no. of pregnancies	21	15	7	5	7	1
Mean maternal age, y (range)	28.8 (23–35)	30.2 (22–37)	31.9 (29–34)	30 (24–34)	31 (28–34)	25
No. of pregnancies with samples in both baseline and all three trimesters—no. (% of total)	8 (38.1)	4 (26.7)	4 (57.1)	1 (20)	2 (28.6)	0
Seizure type—no. (% of total WWE)						
Generalized	18 (85.7)	10 (66.7)	3 (50)	4 (100)	2 (33.3)	0
Focal	3 (14.3)	5 (33.3)	2 (33.3)	0	4 (66.7)	1 (100)
Unclassified	0	0	1 (16.7)	0	0	0
No. of pregnancies with seizure‐free during 9 months before pregnancy, no. (% of total)	15 (71.4)	7 (46.7)	4 (57.1)	3 (60)	3 (42.9)	0
No. of seizure worsening during pregnancy, no. (% of total)	10 (47.6)	8 (53.3)	0	2 (40)	4 (57.1)	1 (100)

Abbreviations: CBZ, carbamazepine; LEV, levetiracetam; LTG, lamotrigine; OXC, oxcarbazepine.

### 3.1 Clearance changes during pregnancy

**FIGURE 2:**
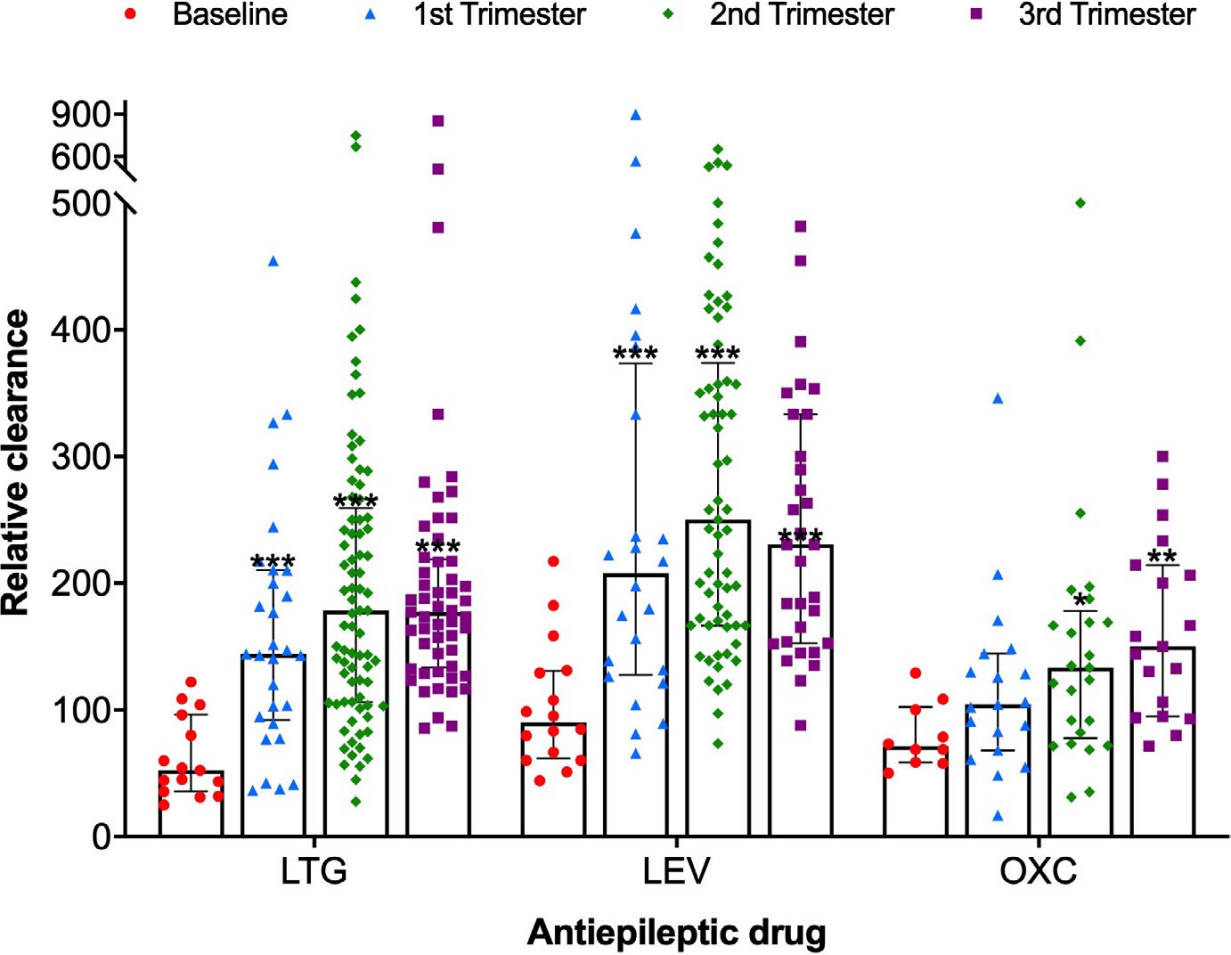
Relative clearance of each antiepileptic drug (AED) during pregnancy. The histogram represented the median with interquartile relative clearance values (daily dose [mg/day]/AEDs concentration [μg/ml]) during baseline (preconception) and each trimester of pregnancy. All clearance values were then logarithm‐transformed to the assumption of Gaussian distribution, and * *p *< 0.05, ** *p *< 0.03, and *** *p *< 0.001 represented significant differences compared with baseline by one‐way ANOVA, respectively

A comparison of relative clearance for each trimester and baseline showed a significant increase in clearance of each AEDs as the pregnancy progressed (Figure 2). The peak clearance of both LTG and LEV occurred in the second trimester during pregnancy, and average peak clearance increased by 3.42‐fold for LTG and 2.78‐fold for LEV from baseline relative clearance (*p *< 0.001), respectively; the relative clearance of LTG was 178.6 L/24 h [An interquartile range (IQR): 106.4, 259.2] compared with a baseline level of 52.2 L/24h (IQR: 35.7, 96.2), and LEV was 250 L/24 h (IQR: 166.7, 373.8) compared with a baseline level of 90 L/24 h (IQR: 61.7, 130.6), respectively; but OXC peak clearance occurred in the third trimester, 2.11‐fold baseline relative clearance (*p *< 0.03), the relative clearance of OXC was 150 L/24 h (IQR: 95.1, 214.3) compared baseline level of 71 L/24 h (IQR: 58.4, 102.1). It was observed that the clearance of LTG, LEV, and OXC increased progressively as the pregnancy processes; however, no significant changes were observed from the first trimester to the third trimester of pregnancy.

### 3.2 AED dose and plasma AED concentration

**FIGURE 3:**
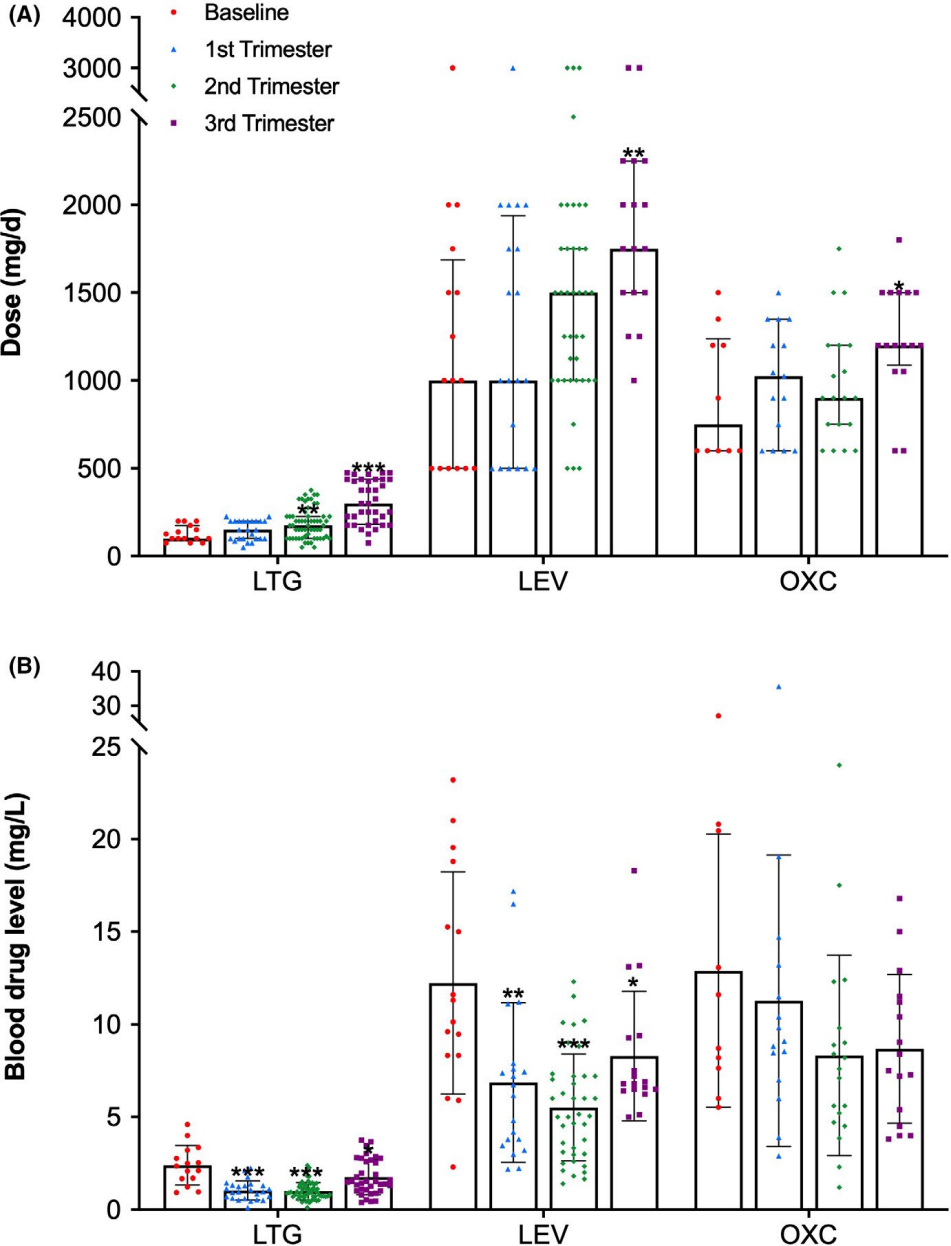
Changes in dose and blood drug levels of AEDs during pregnancy. The histograms represented the median with interquartile of each AED daily dose (A) and mean with standard deviation (SD) of the corresponding blood drug level (B) in each trimester, respectively. * *p *< 0.05, ** *p *< 0.03, and *** *p *< 0.001 represent significant differences compared with baseline using Mann‐Whitney test for dose comparison or a two‐sample *t* test for blood drug level comparison, respectively

The total daily dose of the three AEDs significantly increased as the pregnancy progressed to offset the decreased plasma concentration caused by increased clearance, thereby stabilizing seizures control. The peak dose of all three AEDs occurred in the third trimester of pregnancy (Figure 3). However, their mean blood drug concentrations decreased to varying degrees than the baseline level during every trimester, particularly in the second trimester. And the blood concentrations of LTG, LEV and OXC were lowered by 58.8% (*p *< 0.001, 95% CI [1.05, 1.77]), 54.9% (*p *< 0.001, 95% CI [4.33, 9.11]), and 35.5% (*p *= 0.067, 95% CI [−0.34, 9.49]), respectively, compared with each baseline level.

### 3.3 Therapeutic drug monitoring and seizure frequency during pregnancy

Of all 56 pregnancies, 25 (44.6%) had an increased frequency of seizures at any stage of pregnancy (Table 1). 9 (28.1%) of 32 pregnancies with seizure‐free during the first nine months before pregnancy suffered seizure worsening at any trimester during pregnancy; among them, 6 of LTG monotherapy, 2 of LEV monotherapy, 1 of LEV combined with CBZ polytherapy. In contrast, 16 of 24 (66.7%) pregnancies having any seizures before pregnancy experienced an increased frequency of seizures during pregnancy.

**FIGURE 4:**
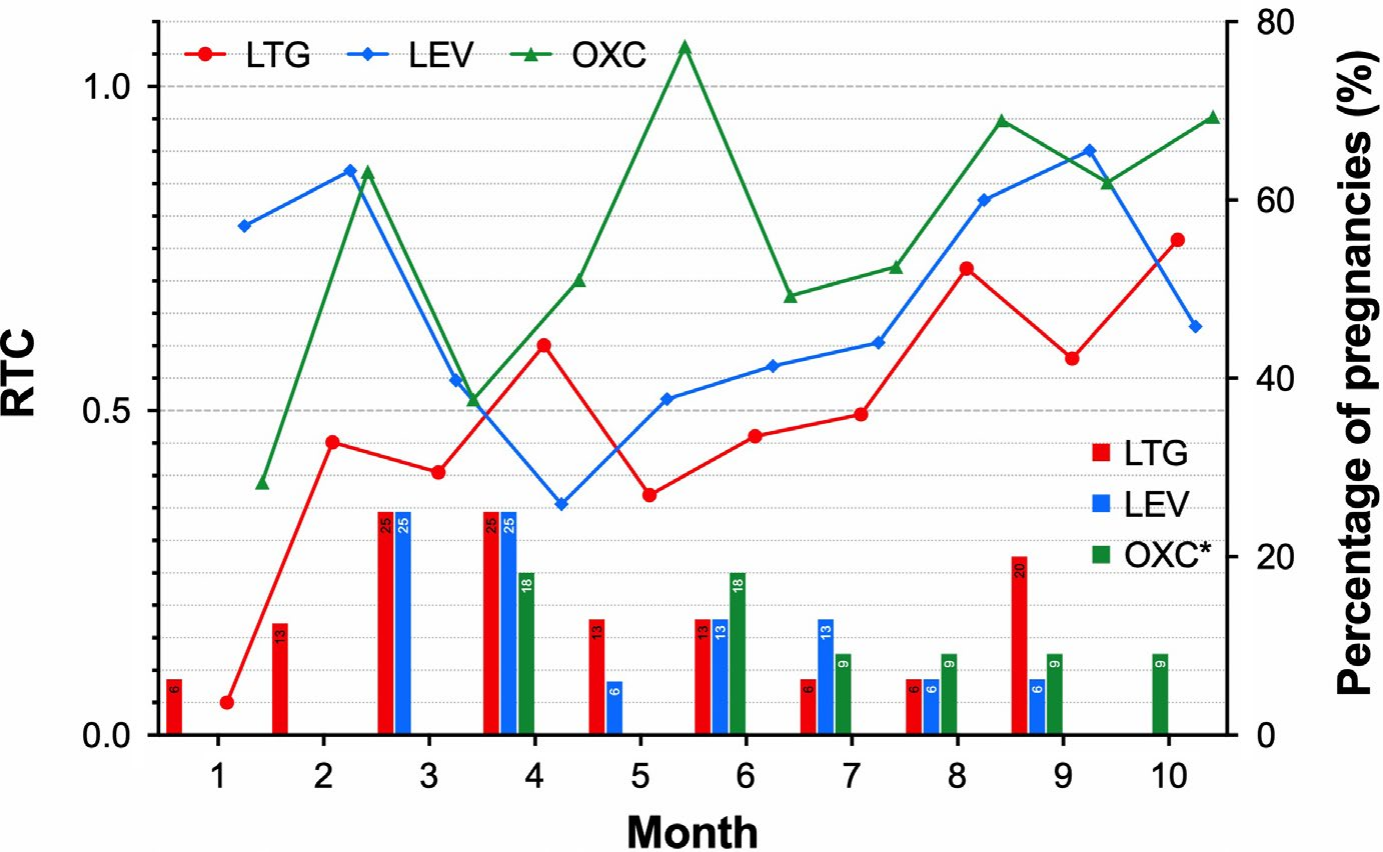
Ratio to target concentration (RTC) with each AEDs and percentage of patients with seizures worsening per month during pregnancy. Line chart depicts changes in RTC values of LTG, LEV, and OXC in different months during pregnancy, and the histogram illustrates the percentage of patients with seizure worsening in the corresponding period. LEV, levetiracetam; LTG, lamotrigine; OXC, oxcarbazepine.* polytherapy of OXC combined LEV

An overview of RTC values of each AED for different months of pregnancy and the percentage of pregnancies with increased frequency of seizure (seizure worsening) compared to nonpregnancy baseline were shown in Figure 4. The results revealed that the highest seizure frequency occurred in the third to fourth months of pregnancy for all three AEDs. As the pregnancy progressed, under the guidance of TDM, the RTC values gradually elevated following the increase in the AED dose, and the seizure frequency decreased.

To clarify the relationship between seizure frequency and fluctuations of AEDs plasma concentration, we compared RTC values for each AED (monotherapy and polytherapy) between patients with and without increased seizures during each trimester irrespectively. The results showed no significant effect of the RTC values on the increased frequency of any seizure types was observed in any trimester, except the RTC value of LTG during the first trimester (Table 2). In addition, no significant differences in seizure worsening of all types were found between patients with >35% decrease in RTC below the nonpregnant baseline and those with less RTC change during pregnancy. Nevertheless, by binary regression analysis, some factors such as RTC values, especially seizures during nine months before pregnancy, could significantly affect seizure frequency during pregnancy. Besides, similar findings were not observed in polytherapy and the ratio to a baseline relative clearance (Table 3).

**TABLE 2:** Comparison of RTC values in women with seizure worsening and those without worsening

Stages of pregnancy	1st Trimester		2nd Trimester		3rd Trimester		Overall	
Increased seizures	Yes	No	Yes	No	Yes	No	Yes	No
LTG samples (*n*)	6	19	9	50	4	32	19	101
Dose [range]	175 [144, 200]	100 [100, 200]	150 [106, 200]	187.5 [100, 238]	237.5 [206, 381]	312.5 [175, 438]	200 [125, 200]	200 [113, 306]
RTC of LTG	0.29 ± 0.11	0.44 ± 0.2	0.50 ± 0.27	0.47 ± 0.25	0.66 ± 0.25	0.67 ± 0.30	0.47 ± 0.26	0.53 ± 0.27
*p* *	0.03		0.78		0.96		0.36	
LEV samples (*n*)	2	18	9	30	3	14	14	62
Dose [range]	1500 [1000, –]	1000 [500,1812]	2000 [1250, 3000]	1250 [1000, 1750]	2250 [1250, –]	1750 [1500, 2062]	2000 [1187, 3000]	1500 [1000, 1750]
RTC of LEV	0.62 ± 0.16	0.71 ± 0.69	0.57 ± 0.22	0.51 ± 0.27	0.7 ± 0.1	0.85 ± 0.28	0.61 ± 0.19	0.64 ± 0.45
*p* *	0.86		0.48		0.38		0.78	
OXC samples (*n*)	0	15	4	15	3	13	7	43
Dose [range]		1025 [600, 1350]	675 [600,975]	900 [750, 1200]	600 [600, ‐]	1200 [1200, 1500]	600 [600, 1050]	1200 [900, 1350]
RTC of OXC	–	1.12 ± 1.52	0.71 ± 0.15	0.82 ± 0.69	0.63 ± 0.2	0.99 ± 0.48	0.68 ± 0.16	0.98 ± 0.91
*p* *	–		0.77		0.23		0.44	

* The *t* test was used to compare the changes of RTC values between patients with seizures worsening and patients without increased seizure frequency.

**TABLE 3:** Binary logistic regression analysis for seizure worsening during pregnancy

Variable	B	S.E.	OR	95%CI		*p*‐value
RTC values	−3.853	1.535	0.02	0.01	0.43	0.012
Maternal age	−0.267	0.083	0.77	0.65	0.9	0.001
Seizure during 9 months before pregnancy	2.198	0.652	9.01	2.5	32.3	0.001
Polytherapy	−0.127	0.623	0.88	0.26	2.98	0.84
Ratio to a baseline (preconception) relative clearance	−0.29	0.182	0.75	0.52	1.07	0.11

Abbreviations: B, unstandardized coefficients; CI, confidence interval; OR, odds ratio; RTC, the ratio of newer AEDs of each trimester to their targets (nonpregnant baseline) concentrations; S.E., standard error.

## 4 DISCUSSION

Previous studies on the changes in the clearance of newer antiepileptic drugs during pregnancy were more limited, particularly for LEV and OXC, and our study extended the available data on the pharmacokinetic changes for newer AED during pregnancy. Our results showed that the clearance of all the three newer AEDs began in the first trimester and increased throughout pregnancy (Figure 2). The most significant clearance changes occurred in the second and third trimester for LTG and in the second trimester for LEV, reaching 3.42‐fold and 2.78‐fold of nonpregnant clearance, respectively, and these findings were consistent with the studies by Reisinger et al.^12^ Besides, significant clearance changes were also seen for OXC, with a mean maximal clearance of 2.11‐fold nonpregnant clearance in the third trimester. However, a prospective study observed that the mean maximal clearance of LEV reached 1.71‐fold baseline clearance in the first trimester while OXC reached a 1.63‐fold baseline clearance in the second trimester,^5^ indicating that the time and degree of clearance changes in newer AEDs during pregnancy differ across individuals. Understanding the characteristics of these pharmacokinetic changes is of particular importance to clinical practice—A timely dose adjustment should be made to offset the significant decrease in AED plasma concentration caused by enhanced drug clearance to lower the risk of seizures.

LTG is a relatively well‐studied second‐generation AED and is widely used as a first‐line agent in WWE of childbearing age.^7^ It is extensively metabolized by uridine‐diphosphate glucuronosyltransferase (UGT) 1A4 and eliminated unchanged by the renal. A noticeable pharmacokinetic change in LTG during pregnancy is attributed to estrogen‐induced glucuronidation and increased renal blood flow,^13^ making it challenging to stabilize plasma levels during pregnancy.^14^ Previous studies have shown that apparent oral clearance of LTG in the second trimester and third trimester almost doubled, and total blood drug concentration decreased by 40%–60%, compared to that of the nonpregnant baseline.^10^, ^15^ In this study, the maximal clearance of LTG increased by 3.42 times, and the mean blood drug levels decreased by 57% in the second trimester (Figures 2 and 3). Pharmacokinetic changes induced by pregnancy are significantly seen in glucuronidated drugs, such as LTG and OXC. OXC is almost completely metabolized to the pharmacologically active metabolite monohydroxycarbazepine after oral intake, which is eliminated by conjugation with glucuronic acid. Similar to LTG clearance, it is reasonable to assume that increased OXC clearance during pregnancy correlates with enhanced hepatic glucuronidation induced by estrogen.^16^ Although a noticeable increase in the clearance of OXC active metabolite was observed in the third trimester of pregnancy in this study, no statistically significant differences in the blood drug levels were found in comparing each trimester versus the baseline levels. Maybe it was due to the broad confidence limited suggesting that variability was an issue. Even so, its blood drug level was still reduced by 35% of baseline levels in the second trimester (Figure 3B), which was in accordance with a 36% decline observed in the previous study.^14^ Furthermore, as one of the most commonly used AEDs in WWE,^17^ LEV also dramatically changed pharmacokinetics during pregnancy. The mean plasma levels of LEV in the second trimester decreased by up to 45.6% compared to baseline levels (Figure 3). About two‐thirds of an oral dose of LEV is excreted unchanged by renal, and some are metabolized by extrahepatic hydrolysis.^18^ Increased renal blood flow may contribute to the dramatic alteration in the apparent oral clearance of LEV during pregnancy.^14^

Several studies have shown a link between increased seizures and the decline in plasma concentrations of AEDs whose metabolism is pronounced altered in pregnancy, for example, LTG, LEV, and OXC,^5^, ^6^, ^19^ but about two‐thirds of WWE remain seizure‐free throughout their pregnancy.^20^ Besides, a previous study has not found a linear relationship between AEDs plasma levels and seizure risks.^12^ As shown in Figure 4 in this study, we observed a trend that a higher percentage of pregnancies with deterioration in seizure control occurred in the pregnancies with low RTC values. Nevertheless, no significant differences in RTC values were observed between pregnancies with and without seizure worsening. Furthermore, our analysis also found that a >35% decrease in AEDs blood levels could not discriminate patients with and without increased seizures during pregnancy, neither for LTG nor the other AEDs, for example, LEV or OXC, differing from previous findings by Pennell et al.^5^, ^6^ which advised a > 35% decrease in AEDs blood levels as a threshold for dose adjustment under TDM during pregnancy. We considered this discrepancy probably correlated with the high proportion of patients with seizure‐free before pregnancy included in our analysis. These patients would have a relatively low risk of seizure worsening, even if a significant fluctuation in blood AEDs concentrations existed during pregnancy. In addition, a smaller sample size and retrospective study design might have weakened the connection between RTC decrease and seizure deterioration. However, we should recognize the fact that the risk factors leading to seizure worsening during pregnancy are multiplicity except for a precipitous decline in the levels of some newer AEDs during pregnancy; other critical pregnancy‐related factors can also contribute to seizure deterioration, including hormone fluctuations, sleep cycle disorders, and stress.^21^ Our data also showed that seizure deterioration occurred primarily in the first trimester during pregnancy, partly be attributed to dose changes that did not keep up with changes in blood drug level (Figures 3 and 4). However, during the second and third trimesters, AED dosage increased significantly under the guidance of TDM, accompanied by a reduction in the percentage of pregnancies with seizure deterioration (Figure 3).

We found some risk factors that could significantly affect seizures of all types during pregnancy, including the RTC values, maternal age, especially seizures during nine months before pregnancy through binary logistic regression analysis. As we know, the absence of seizures in the preconception period (usually at least nine months) is a strong predictor of remaining seizure‐free during pregnancy.^22^ Similar results have been observed in our study; that is, patients with poor seizure control before pregnancy were 9 times more susceptible to increased seizure frequency during pregnancy than those with seizure‐free or seizure stability before pregnancy (OR: 9.01, CI: 2.5–32.3, *p *= 0.001). In addition, no evident differences in RTC values were observed between patients with and without increased seizures during pregnancy. However, regression analysis still showed that RTC values seemed to have an effect on seizure control during pregnancy (OR: 0.02, CI: 0.01–0.43, *p *= 0.012). Intriguingly, the results also showed that maternal age seemed to be negatively correlated with seizure aggravation during pregnancy (OR: 0.77, CI: 0.65–0.9, *p *= 0.001), indicating a younger WWE was more likely to have a higher risk of worsening seizure during pregnancy. This finding was consistent with the result of a study from 841 pregnancies in the Australian Register of Antiepileptic Drug Treated Pregnancies, which showed that younger mothers were more commonly seen in seizure affected pregnancies than in seizure‐free pregnancies and were more likely to have an earlier epilepsy onset and a longer epilepsy duration shortening seizure‐free periods before pregnancy.^23^

We should notice several limitations of this retrospective study. First, three newer AEDs and a small sample size limited our ability to utilize the findings to the majority of the WWE population. However, these data were also a substantial extension to the limited information available; in particular, there were fewer pieces of literature available on the pharmacology of LEV and OXC. Second, the clinical data did not include a well‐documented record of patient's compliance and we could not exclude the possibility that some blood samples might not be consistent with the actual AEDs dosage. Third, as we know, monotherapy is preferred above polytherapy to investigate the pharmacokinetic characteristics of AEDs because it provides a better insight into the specific effects of AEDs during pregnancy. Nevertheless, because LEV has a low plasma‐protein binding and fewer drug‐drug interactions with LTG and OXC, in our analysis, we enrolled pregnancies with polytherapy combined with LEV, which is not thought to affect the pharmacokinetics of LTG and OXC and vice versa.^9^, ^12^, ^24^ Finally, we did not consider the effect of genetic polymorphisms of UGT on the AEDs blood levels in WWE, as more minor changes in the pharmacokinetics of LTG in the third trimester have been observed in women with UGT1A4 142TG (*3) polymorphism than those with wild type.^25^ Future works should cover an extensive sample size and well‐designed prospective studies on newer AEDs to clarify the association of fluctuation of plasma AED concentration with deterioration of seizure control and further investigate the role of TDM on seizure control during pregnancy.

## 5 CONCLUSIONS

Newer AEDs, including LTG, LEV, and OXC, have a pronounced increase in apparent oral clearance and decreased plasma concentration during pregnancy. TDM monitoring of these newer AEDs is important for poorly controlled and well‐controlled patients. We would encourage at least one monitoring every trimester and probably more frequently for women with poorly seizure control before pregnancy, and dose adjustment should keep up with clearance changes that cannot be predicted prospectively. Moreover, given the considerable variability of risk factors associated with deterioration of seizure control during pregnancy, we should also focus on pre‐pregnancy counseling and seizure control before pregnancy for WWE.

## CONFLICT OF INTERESTS

All authors declare that they have no conflicts of interest.
